# Quercetin Mediated Salt Tolerance in Tomato through the Enhancement of Plant Antioxidant Defense and Glyoxalase Systems

**DOI:** 10.3390/plants8080247

**Published:** 2019-07-25

**Authors:** Khursheda Parvin, Mirza Hasanuzzaman, M. H. M. Borhannuddin Bhuyan, Sayed Mohammad Mohsin, Masayuki Fujita

**Affiliations:** 1 Laboratory of Plant Stress Responses, Department of Applied Biological Science, Faculty of Agriculture, Kagawa University, Miki-Cho, Kita-Gun, Kagawa 761-0795, Japan; 2 Department of Horticulture, Sher-e-Bangla Agricultural University, Sher-e-Bangla Nagar, Dhaka 1207, Bangladesh; 3 Department of Agronomy, Sher-e-Bangla Agricultural University, Sher-e-Bangla Nagar, Dhaka 1207, Bangladesh; 4 Citrus Research Station, Bangladesh Agricultural Research Institute, Jaintapur, Sylhet 3156, Bangladesh; 5 Department of Plant Pathology, Sher-e-Bangla Agricultural University, Sher-e-Bangla Nagar, Dhaka 1207, Bangladesh

**Keywords:** reactive oxygen species, cellular damage, methylglyoxal, phenolic compounds

## Abstract

Quercetin (Qu) is a strong antioxidant among the phenolic compounds having physiological and biochemical roles in plants. Hence, we have studied the Qu evolved protection against salinity in tomato (*Solanum lycopersicum* L.). Salinity caused ionic toxicity by increasing Na^+^ content in seedlings along with nutritional starvation of K^+^, Ca^2+^, and Mg^2+^. While osmotic stress was detected by higher free proline (Pro) content and lower leaf relative water content (LRWC) in salt-stressed seedlings. Salt toxicity also induced higher H_2_O_2_ generation, malondialdehyde (MDA) content and lipoxygenase (LOX) activity as a sign of oxidative stress. Tomato seedlings suffered from methylglyoxal (MG) toxicity, degradation of chlorophyll, along with lower biomass accumulation and growth due to salt exposure. However, Qu application under salinity resulted in lower Na^+^/K^+^ due to reduced Na^+^ content, higher LRWC, increased Pro, and reduction of H_2_O_2_ and MDA content, and LOX activity, which indicated alleviation of ionic, osmotic, and oxidative stress respectively. Quercetin caused oxidative stress, lessening through the strengthening of both enzymatic and non-enzymatic antioxidants. In addition, Qu increased glutathione *S*-transferase activity in salt-invaded seedlings, which might be stimulated reactive oxygen species (ROS) scavenging along with higher GSH content. As a result, toxic MG was detoxified in Qu supplemented salt-stressed seedlings by increasing both Gly I and Gly II activities. Moreover, Qu insisted on better plant growth and photosynthetic pigments synthesis in saline or without saline media. Therefore, exogenous applied Qu may become an important actor to minimize salt-induced toxicity in crops.

## 1. Introduction

Salinity is a major abiotic constraint for limiting plant growth; physiology and development lead to yield loss [1]. Under salinity, major secondary stresses—including nutrient deficit, osmotic, and oxidative stress—occur in plants.

At early reaction, salt stress causes higher water potential in the root zone and restricts water uptake causing osmotic stress. Consequently, plants suffer from nutrients deficiency due to limited nutrient uptake, as well as the transport to growing parts [1]. Moreover, the plant suffers from ionic stress due to increasing Na^+^/K^+^ ratio with higher Na^+^ influx and K^+^ efflux [2,3]. Additionally, excess Na^+^ causes chlorosis and necrosis in leaves along with premature senescence [2,3]. Therefore, plants try to avoid osmotic and ionic stress through various mechanisms including osmotic adjustment, limiting the cell expansion and cell division, and regulation of stomatal movement. As a result, it reduces the leaf area, which lowers photosynthesis as well as decreasing plant growth [1,2,3]. Salinity is also responsible for the overproduction of reactive oxygen species (ROS: singlet oxygen, ^1^O_2_; superoxide, O_2_^•−^; hydrogen peroxide, H_2_O_2_; hydroxyl radical, OH^•^) and thus induces oxidative stress in plants [3]. Plants suffer from oxidative stress from the damage of cellular organelles—including lipid, protein, and nucleic acid—as well as disorganizing the cellular membrane [4]. Plants respond to the stress stimuli and transmit signals for physiological and biochemical changes immediately after stress exposure for endurance and growth [5]. Thus, produced secondary metabolites regulate plant physiological processes to manage salt-induced stress [6]. Subsequently, the plants should regulate ROS production to check injurious effects along with securing signaling functions [4]. In this aspect, the antioxidant defense system is very much efficient to detoxify overproduced ROS, which consists of enzymatic (superoxide dismutase, SOD; catalase, CAT; ascorbate peroxidase, APX; monodehydroascorbate reductase, MDHAR; dehydroascorbate reductase, DHAR; glutathione reductase, GR; glutathione peroxidase, GPX; and glutathione *S*-transferase, GST) and non-enzymatic components (ascorbate, AsA; glutathione, GSH; carotenoids, phenolic compounds, alkaloids, flavanones, anthocyanins, etc.) to smoothen the ROS detoxification process systematically [4]. Additionally, stress alters methylglyoxal (MG) synthesis, which at lower concentration acts as a signaling molecule, and regulates cell redox homeostasis. However, stress-induced elevated MG causes toxicity and triggers ROS production [7]. Hence, the efficient MG detoxification became an important biochemical marker for stress tolerance, where GSH dependent glyoxalase pathway efficiently perform MG detoxification via glyoxalase I (Gly I) and glyoxalase II (Gly II) enzymes [4].

Reports suggested that genetic self-defense capacity is not enough to fully secure the plants from oxidative damage in most of the cases. Therefore, enhancing salinity tolerance by upregulating the antioxidant defense and glyoxalase systems through the use of chemicals as a protectant from the exogenous source has recently become quite popular. Researchers are currently testing diverse groups of chemicals including phytohormone, organic acid, essential nutrient molecules, antioxidants, and other plant-derived secondary metabolites as phytoprotectants. Among secondary metabolites, many phenolic compounds have been reported to confer stress tolerance [8]; especially flavonoids and phenolic acids, which are produced from the shikimate-phenylpropanoid biosynthetic pathway, and the most widespread subgroup [9]. Higher accumulations of phenolic compounds scavenge ROS and act as antioxidants to protect the plant from stress-induced injury [10,11]. Hence, quercetin (Qu) is one of the flavonols, which is a subgroup of flavonoids found mainly in the form of glycosides in fruits and vegetable plants and rarely found in aglycon form [10,12]. Quercetin has free radical scavenging and antioxidant ability, along with anti-inflammatory and anti-carcinogenic properties [13,14]. It is already well established that exogenous Qu application reduces oxidative stress in animal systems [15]. Regarding plant systems, reports on Qu-induced oxidative damage mitigation are scant and disorganized. Sánchez-Rodríguez et al. [12] reported that drought-resistant tomato showed higher endogenous accumulation of Qu compared to sensitive cultivars. Moreover, supplemental Qu protects *Arabidopsis* from paraquat toxicity by chlorophyll (Chl) and protein stabilization [16]; which inspired and demonstrated the possibility and scope to test Qu exogenously as a salinity protector. Moreover, it is also essential to study exogenous Qu-induced regulation of antioxidant defense system under salinity.

Therefore, we investigated the response of exogenous Qu-treated tomato seedlings upon salt toxicity considering morphological, physiological, and biochemical attributes. In addition, Qu-mediated antioxidant defense, glyoxalase systems, and mineral homeostasis were also investigated. To the best of our knowledge, this is the first report to elucidate exogenous Qu-induced salt stress tolerance in tomato, where the coordinated actions of antioxidant defense, glyoxalase system, and mineral homeostasis were addressed together.

## 2. Results

### 2.1. Quercetin Improved Plant Growth and Biomass Production under Salt Stress

**Figure 1:**
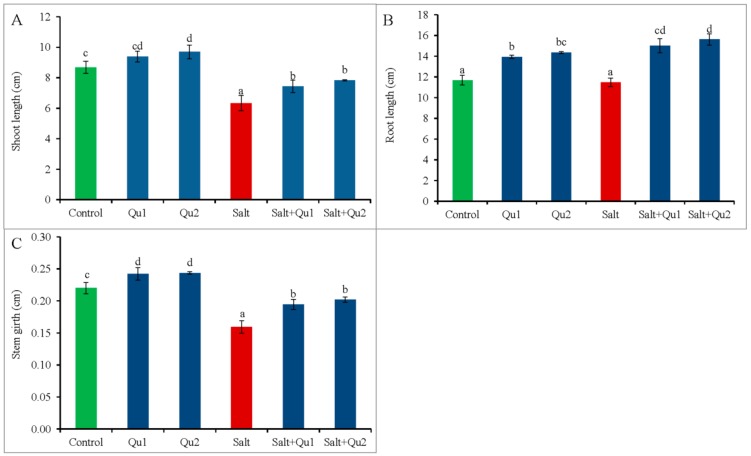
Morphological growth (**A**: shoot length; **B**: root length; and **C**: stem girth) in tomato seedlings treated with salt (150 mM NaCl) with and/or without quercetin (Qu1; 15 µM and Qu1; 25 µM) for 5 days. Three experimental replications (n = 3) were used to calculate mean (±SD), where each treatment contained a total of 10 seedlings per replicate. Statistically different values are shown by dissimilar letters (*p* ≤ 0.05; Fisher’s LSD test).

**Figure 2:**
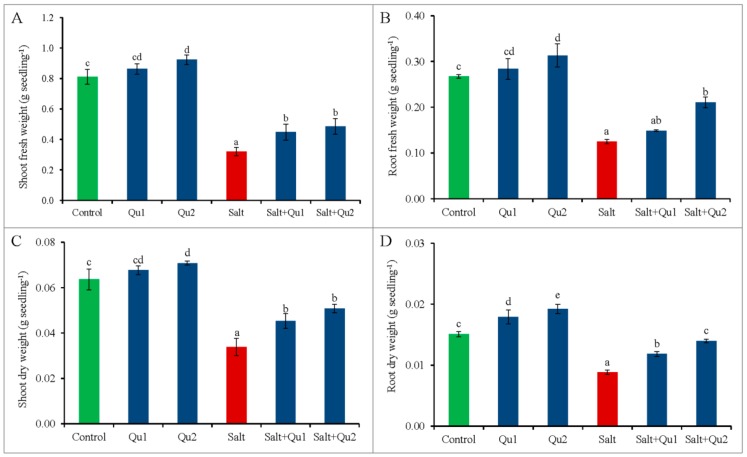
Seedlings fresh and dry weight (**A**: shoot fresh weight; **B**: root fresh weight; **C**: shoot dry weight; and **D**: root dry weight) in tomato seedlings treated with salt (150 mM NaCl) with and/or without quercetin (Qu1; 15 µM and Qu2; 25 µM) for 5 days. Three experimental replications (n = 3) were used to calculate mean (±SD), where each treatment contained a total of 10 seedlings per replicate. Statistically different values are shown by dissimilar letters (*p* ≤ 0.05; Fisher’s LSD test).

We have selected two doses of Qu by observing the better response from seedlings after conducting several trail experiments consisting of various doses of Qu against 150 mM of NaCl. Salt-stress reduced tomato seedling growth confirmed from lowering of shoot length, root length, and stem girth, compared to control (Figure 1A–C); while salt-induced lower biomass accumulation was indicated by reduced fresh weight (FW) and dry weight (DW) of both shoots and roots (Figure 2A–D). Results indicate that exogenous Qu supplementation improved seedling growth and biomass accumulation under both saline and non-saline conditions (Figure 1A–C and Figure 2A–D), where 25 µM Qu showed the best growth healing except for root FW and DW, which was best from 15 µM Qu supplementation under both stressed and non-stressed conditions.

**Figure 3:**
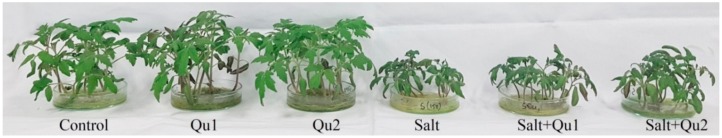
Visual differences of tomato seedlings treated with salt (150 mM NaCl) with and/or without quercetin (Qu1; 15 µM and Qu2; 25 µM) for 5 days.

Afterward, exogenous Qu induced growth improvement, as well as salt tolerance, was also visualized from the phenotypic appearance (Figure 3).

### 2.2. Quercetin Maintained Photosynthetic Pigments Contents under Salinity

**Figure 4:**
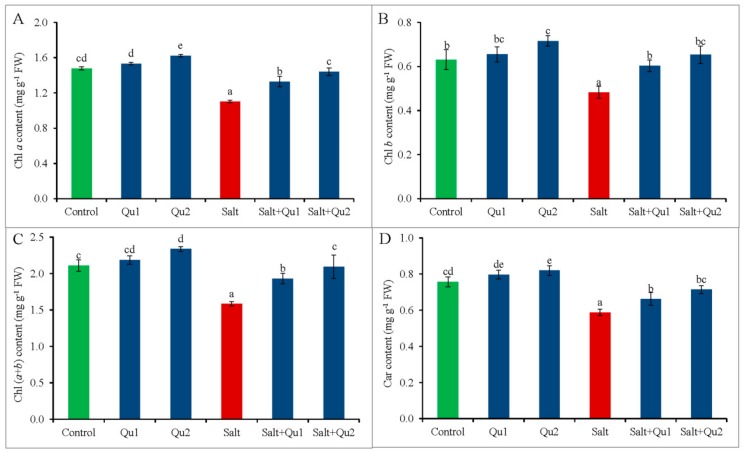
Photosynthetic pigments content including Chl *a* content (**A**); Chl *b* content (**B**); Chl (*a* + *b*) content (**C**); and carotenoid content (**D**) in tomato seedlings treated with salt (150 mM NaCl) with and/or without quercetin (Qu1; 15 µM and Qu2; 25 µM) for 5 days. Three experimental replications (n = 3) were used to calculate mean (±SD), where each treatment contained a total of 10 seedlings per replicate. Statistically different values are shown by dissimilar letters (*p* ≤ 0.05; Fisher’s LSD test).

Salinity decreased the contents of photosynthetic pigments indicated by lower Chl *a*, Chl *b*, and Chl (*a* + *b*) and carotenoid (Car) contents (Figure 4A–D). However, Qu supplementation improved Chl *a*, Chl *b*, and Chl (*a* + *b*) and Car contents in seedlings under both salt stress and control conditions. Compared to stressed treatment alone, 25 µM Qu application showed a maximum increase of Chl *a*, Chl *b*, and Chl (*a* + *b*) along with higher Car content in salt-stressed seedlings (Figure 4A–D).

### 2.3. Quercetin Mediated Na^+^/K^+^ Homeostasis under Salinity

**Figure 5:**
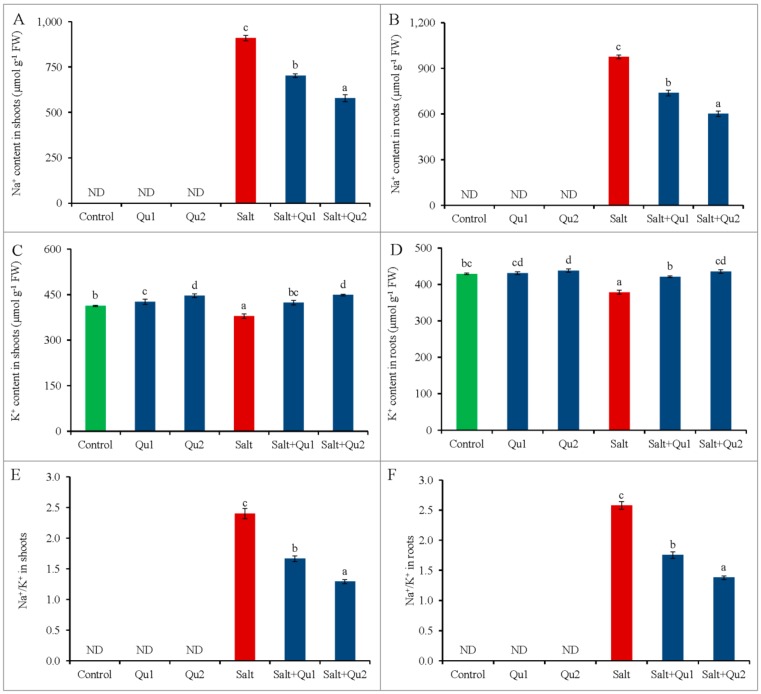
Content of Na^+^ and K^+^ (**A**: shoot Na^+^ content; **B**: root Na^+^ content; **C**: shoot K^+^ content; **D**: root K^+^ content; **E**: shoot Na^+^/K^+^; and **F**: root Na^+^/K^+^) in tomato seedlings treated with salt (150 mM NaCl) with and/or without quercetin (Qu1; 15 µM and Qu2; 25 µM) for 5 days. Three experimental replications (n = 3) were used to calculate mean (±SD), where each treatment contained a total of 10 seedlings per replicate. Statistically different values are shown by dissimilar letters (*p* ≤ 0.05; Fisher’s LSD test).

Salt invaded tomato seedlings showed eminent Na^+^ accumulation in both shoots and roots compared to control (Figure 5A,B) while K^+^ accumulation was reduced in salt-treated plants, compared to control (Figure 5C,D). Thus, salinity increased Na^+^/K^+^ ratio in both shoots and roots, compared to non-saline treatment (Figure 5E,F).

However, Qu feeding suppressed Na^+^/K^+^ ratio (Figure 5E,F) by decreasing Na^+^ accumulation (Figure 5A,B) with maintaining higher K^+^ content (Figure 5C,D) in salt-treated seedlings, compared to the salt-stressed seedlings alone. The lowest Na^+^ accumulation in both shoots and roots was demonstrated in 25 µM Qu applied salt-stressed seedlings with the highest K^+^ content, compared to salt-stressed seedlings alone.

### 2.4. Quercetin Induced Improvement of Nutrient Status under Salinity

**Figure 6:**
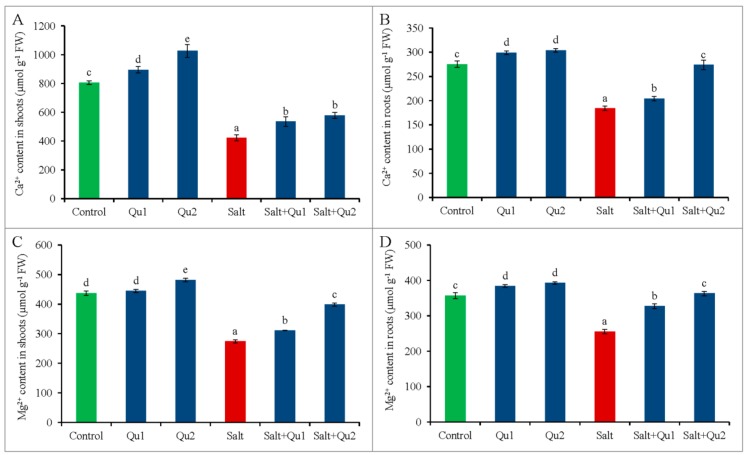
Mineral nutrient status (**A**: shoot Ca^2+^ content; **B**: root Ca^2+^ content; **C**: shoot Mg^2+^content; **D**: root Mg^2+^ content) in tomato seedlings treated with salt (150 mM NaCl) with and/or without quercetin (Qu1; 15 µM and Qu2; 25 µM) for 5 days. Three experimental replications (n = 3) were used to calculate mean (±SD), where each treatment contained a total of 10 seedlings per replicate. Statistically different values are shown by dissimilar letters (*p* ≤ 0.05; Fisher’s LSD test).

Salinity hampered mineral nutrient contents in tomato seedlings by reducing Ca^2+^ and Mg^2+^ in both shoots and roots (Figure 6A–D). Around 48% and 37% reduction of Ca^2+^ and Mg^2+^ contents, respectively, were noticed in 150 mM salt-stressed seedling shoots while roots showed 33% and 28% reduction in Ca^2+^ and Mg^2+^ contents, respectively, compared to control. Meanwhile, Qu application significantly enhanced both Ca^2+^ and Mg^2+^ contents in salt exposed seedlings, compared to only stressed one (Figure 6A–D). Not only under stress conditions, Qu also improved Ca^2+^ and Mg^2+^ content in non-saline conditions. Exogenous Qu-feeding (25 µM) to stressed seedling increased about 37% Ca^2+^ and 45% Mg^2+^ in shoots tissue, while 49% Ca^2+^ and 42% Mg^2+^ in root tissue, respectively, compared to stressed seedlings alone.

### 2.5. Quercetin-Induced Osmotic Status under Salinity

**Figure 7:**
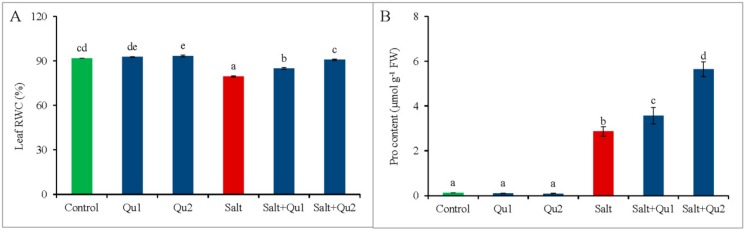
Osmotic stress marker (**A**: leaf RWC and **B**: Pro content) in tomato seedlings treated with salt (150 mM NaCl) with and/or without quercetin (Qu1; 15 µM and Qu2; 25 µM) for 5 days. Three experimental replications (n = 3) were used to calculate mean (±SD), where each treatment contained a total of 10 seedlings per replicate. Statistically different values are shown by dissimilar letters (*p* ≤ 0.05; Fisher’s LSD test).

Tomato seedlings suffered from osmotic stress upon salt exposure, which was confirmed by the reduction of leaf relative water content (LRWC) with elevated free proline (Pro) accumulation (Figure 7A,B). Interestingly, Qu application showed a further increase of Pro accumulation in stressed seedlings along with improved LRWC, compared to stressed-seedlings without Qu supplementation (Figure 7A,B). In contrast to salinity, 25 µM Qu was best to improve osmotic status by increasing about 97% Pro content with better LRWC in salt-treated seedlings.

### 2.6. Quercetin Lessened Salinity-Induced Oxidative Stress

**Figure 8:**
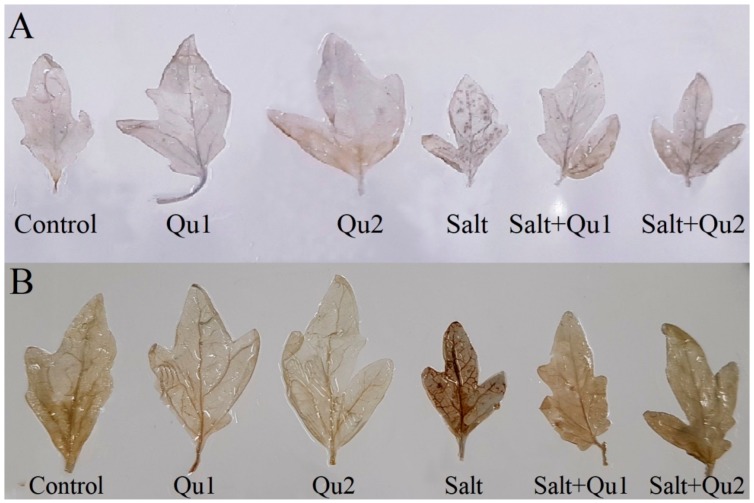
Histochemical detection of oxidative stress markers (**A**: O_2_^•−^ and **B**: H_2_O_2_) in tomato seedlings treated with salt (150 mM NaCl) with and/or without quercetin (Qu1; 15 µM and Qu2; 25 µM) for 5 days.

From histochemical staining, clear blue and brown spots were observed induced by O_2_^•−^ and H_2_O_2_, respectively, which showed the salt-induced oxidative stress in tomato seedlings in comparison with control (Figure 8A,B). However, interestingly, Qu co-treatment along with salinity showed lesser spots for both O_2_^•−^ and H_2_O_2_, compared to stressed treatment alone (Figure 8A,B).

**Figure 9:**
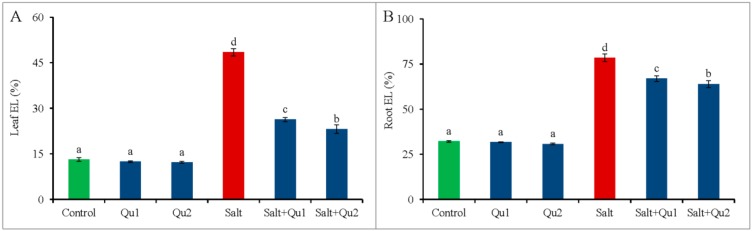
Electrolyte leakage % (**A**: leaf EL; **B**: root EL) in tomato seedlings treated with salt (150 mM NaCl) with and/or without quercetin (Qu1; 15 µM and Qu2; 25 µM) for 5 days. Three experimental replications (n = 3) were used to calculate mean (±SD), where each treatment contained a total of 10 seedlings per replicate. Statistically different values are shown by dissimilar letters (*p* ≤ 0.05; Fisher’s LSD test).

Salinity increased electrolyte leakage (EL) in both leaves and roots of tomato seedlings (Figure 9A,B). However, the lowest EL was observed in non-stressed seedlings with or without Qu supplementation. Moreover, the addition of Qu to salt-stressed seedlings decreased EL in both leaves and roots compared to salt-treated seedlings alone (Figure 9A,B), where maximum EL reduction was observed in 25 µM Qu supplementation in salt-stressed seedlings compared to salt stress alone.

**Figure 10:**
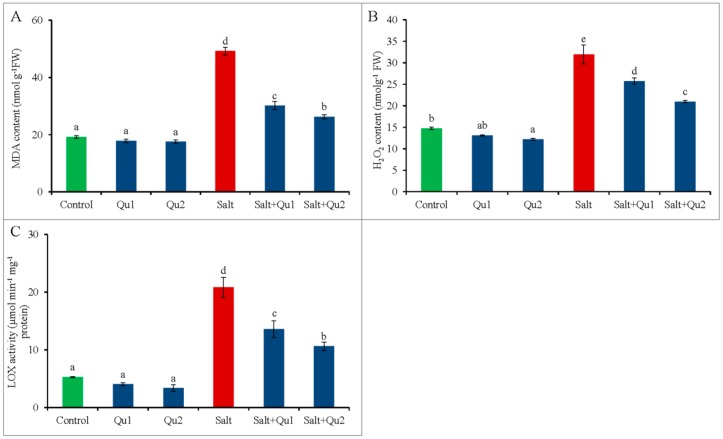
Detection of oxidative stress marker (**A**: MDA content; **B**: H_2_O_2_ content; and **C**: LOX activity) in tomato seedlings treated with salt (150 mM NaCl) with and/or without quercetin (Qu1; 15 µM and Qu2; 25 µM) for 5 days. Three experimental replications (n = 3) were used to calculate mean (±SD), where each treatment contained a total of 10 seedlings per replicate. Statistically different values are shown by dissimilar letters (*p* ≤ 0.05; Fisher’s LSD test).

The H_2_O_2_ content and lipoxygenase (LOX) activity augmented along with elevated malondialdehyde (MDA) content in salt-treated tomato seedlings (Figure 10A–C). In contrast, Qu supplementation reduced H_2_O_2_, MDA contents, and LOX activity. The highest reduction of H_2_O_2_, MDA contents, and LOX activity were observed from 25 µM Qu supplemented salt-stressed seedlings relative to only salt stress (Figure 10A–C).

### 2.7. Quercetin-Mediated Upregulation of Plant Antioxidant Defense System under Salinity

#### 2.7.1. Non-Enzymatic Antioxidants

**Figure 11:**
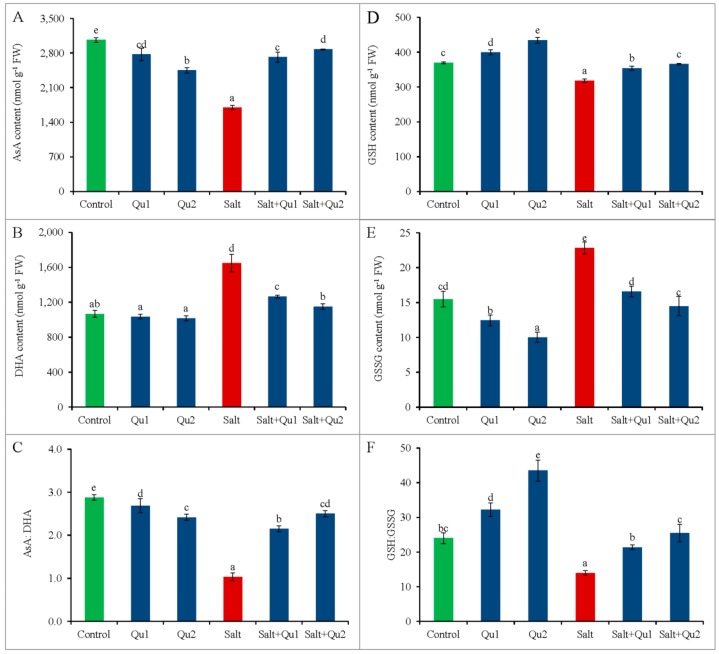
Content of AsA (**A**), DHA (**B**), GSH (**D**), GSSG (**E**); and ratio of AsA/DHA ratio (**C**) and GSH/GSSH (**F**) in tomato seedlings treated with salt (150 mM NaCl) with and/or without quercetin (Qu1; 15 µM and Qu2; 25 µM) for 5 days. Three experimental replications (n = 3) were used to calculate mean (±SD), where each treatment contained a total of 10 seedlings per replicate. Statistically different values are shown by dissimilar letters (*p* ≤ 0.05; Fisher’s LSD test).

Salt stress decreased the AsA content while DHA content increased, resulting in a reduction of AsA/DHA ratio compared to control (Figure 11A–C). In addition, gradual increase of Qu supplementation in non-stressed seedlings showed a sharp reduction of AsA/DHA ratio along with lower AsA content compared to the untreated seedlings. Conversely, Qu addition in salt-stressed seedlings increased AsA/DHA ratio compared with salt-stressed seedlings alone (Figure 11C), where the highest increase in AsA/DHA was found from 25 µM Qu supplementation under salt treatment, while AsA content increased by 69% and DHA content decreased by 30%, compared to the salt-treated seedlings without Qu (Figure 1A–C).

Compared to control, GSH content reduced along with the increment of GSSG content, which resulted in lower GSH/GSSG ratio in salt exposed seedlings (Figure 11D–F). However, Qu feeding improved the GSH/GSSG ratio significantly in salt-stressed seedlings compared to only salt treatment. Hence, 25 µM Qu showed the best result in terms of GSH content increment and GSSG content reduction along with improving GSH/GSSH pair in salt-stressed seedlings compared with salt treatment only (Figure 11D–F). 

#### 2.7.2. Enzymatic Antioxidants

**Figure 12:**
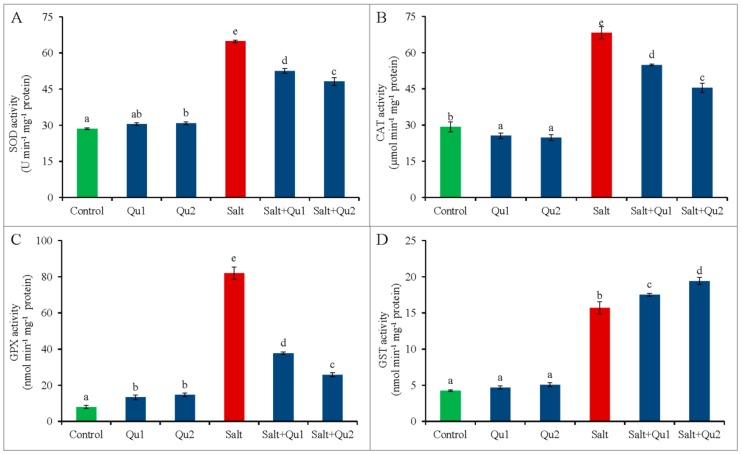
Activities of SOD (**A**), CAT (**B**), GPX (**C**), and GST (**D**) in tomato seedlings treated with salt (150 mM NaCl) with and/or without quercetin (Qu1; 15 µM and Qu2; 25 µM) for 5 days. Three experimental replications (n = 3) were used to calculate mean (±SD), where each treatment contained a total of 10 seedlings per replicate. Statistically different values are shown by dissimilar letters (*p* ≤ 0.05; Fisher’s LSD test).

Tomato seedlings showed a significant increase in SOD activity upon salinity treatment than that of control. Conversely, Qu application to salt-stressed seedlings caused the reduction of SOD activity in parallel with only stressed treatment. Hence, 25 µM Qu application showed the highest reduction of SOD activity in stressed seedlings comparison with only salt-treated one (Figure 12A).

Consequently, increased CAT activity was observed in tomato seedlings, compared to control. However, Qu supplementation reduced CAT activity in stressed seedlings in relative to salt-stressed seedlings alone (Figure 12B).

In line, the GPX activity increased in salt-treated seedlings, compared to control. However, the addition of Qu reduced GPX activity in salt-stressed seedlings than the salinity treatment alone (Figure 12C).

Moreover, tomato seedlings showed increased GST activity under salinity, while Qu application further increased GST activity under salt-treatment compared with the salt-treatment alone (Figure 12D).

**Figure 13:**
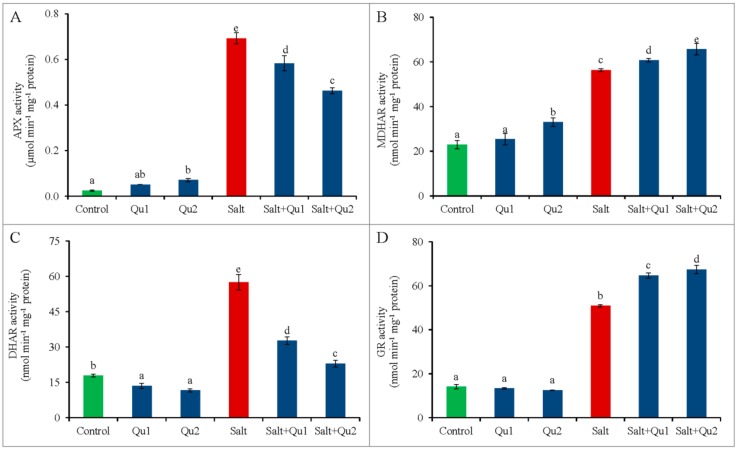
Activities of APX (**A**), MDHAR (**B**), DHAR (**C**), and GR (**D**), in tomato seedlings treated with salt (150 mM NaCl) with and/or without quercetin (Qu1; 15 µM and Qu2; 25 µM) for 5 days. Three experimental replications (n = 3) were used to calculate mean (±SD), where each treatment contained a total of 10 seedlings per replicate. Statistically different values are shown by dissimilar letters (*p* ≤ 0.05; Fisher’s LSD test).

In the AsA-GSH pathway, salt stress increased APX activity. Again, exogenous Qu application decreased the APX activity in stressed seedlings (Figure 13A); whereas the MDHAR activity also increased upon salt exposure, which was further increased by Qu addition (Figure 13B). On the other hand, salt stress enhanced DHAR activity, but 25 µM Qu supplemented salt-stressed seedlings exhibited reduced DHAR activity by 60% (Figure 13C). Again, GR activity increased by 37% in salt-stressed tomato seedlings, compared to control; but the addition of Qu to salt-stressed seedlings further improved GR activity, compared to salt treatment only (Figure 13D).

### 2.8. Quercetin Detoxified Methylglyoxal by Activating Glyoxalase System under Salinity

**Figure 14:**
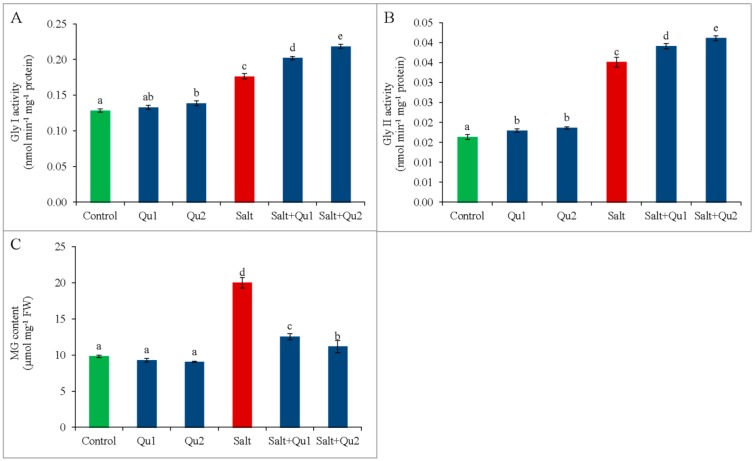
Activities of glyoxalase enzymes (**A**: Gly I and **B**: Gly II) and methylglyoxal content (**C**) in tomato seedlings treated with salt (150 mM NaCl) with and/or without quercetin (Qu1; 15 µM and Qu2; 25 µM) for 5 days. Three experimental replications (n = 3) were used to calculate mean (±SD), where each treatment contained a total of 10 seedlings per replicate. Statistically different values are shown by dissimilar letters (*p* ≤ 0.05; Fisher’s LSD test).

Salt stress increased MG accumulation in tomato seedlings together with glyoxalase (Gly I; and Gly II) activities (Figure 14A–C). However, salt-treated seedlings with 25 µM Qu addition decreased MG content comparison with salt-stressed seedlings alone, most where both Gly I and Gly II activities were further accelerated (Figure 14A–C).

## 3. Discussion

Exogenous uses of different phytoprotectants are very promising due to cost-effectiveness, compared to traditional breeding or transgenic approaches for enhancing plant tolerance. In this section, we will discuss and explain how Qu supplementation alleviated the adverse effects of salinity in tomato seedlings.

Salinity hinders plant growth as a common phenomenon. Salt-induced decrease of seedlings growth with lower biomass accumulation might be due to stress-forced inhibition of cell elongation and cell division [17]. Similarly, reduction of dry weight was also found in stressed tomato seedlings [18]. Saline-induced disturbance of ion homeostasis, osmotic, and oxidative status causes growth reduction [2]. However, the addition of Qu to salt-stressed tomato seedlings recovered from the detrimental effect of salt-stress. Recently, Saleh and Madany [19] found phenolic compound—coumarin (COU)—induced growth improvement in wheat under salinity. Another phenolic compound apigenin also increased growth and dry matter content in salt-stressed rice seedlings [20].

Not only growth parameters but also photosynthetic pigments (Chl *a*, Chl *b*, Chl (*a* + *b*) and Car) are also destroyed keeping relation with oxidative damage in tomato seedlings causing chlorosis. A similar loss of photosynthetic pigments was reported by Ahmed et al. [17] and Martinez et al. [18] in salt-stressed tomato. As an explanation, it was reported that both ionic and oxidative stresses are responsible for Chl degradation due to ROS-induced higher chlorophyllase activity under salinity [2]. In addition, Fatma et al. [21] reported that salinity inhibited photosynthesis by hampering RuBisCo biosynthesis, Chl biosynthesis, as well as photosystem I activity. Interestingly, Qu supplementation enhanced the status of photosynthetic pigments in salt-stressed tomato seedlings, which might be due to Qu-induced lower Na^+^/K^+^ ratio, ROS scavenging and alleviation of osmotic stress. Likely, apigenin also improved Chl and Car synthesis in salt-stressed rice [20]. Araniti et al. [22] also reported that cinnamic acid—a potent phenolic compound—increased Chl *a*, Chl *b*, and Car contents in maize seedlings.

Salt stress disrupts the Na^+^/K^+^ balance in cells by accumulating excess Na^+^ with lesser K^+^ [2]. Moreover, essential nutrient assimilation was also disrupted due to membrane selectivity and/or competitive interactions among the ions, which resulted in alteration of nutrient metabolism [3,23]. In the present study, we also found increased Na^+^/K^+^ ratio in both shoots and roots due to higher Na^+^ content and lower K^+^ content, which broke the ionic balance. Recently, Zhou et al. [23] reported similar results in tomato under salinity. Assimilation of excess Na^+^ by roots and subsequent transportation to shoots cause nutrient starvations by reducing uptake, transportation, and accumulation of other minerals including K^+^, Ca^2+^, and Mg^2+^ [3]. Therefore, maintaining intracellular ionic balance is one of the most important salt tolerance mechanisms for the plant. Moreover, ion balancing is the reflection of cellular stability as well as the prerequisite of cell health for controlling normal physiological and biochemical processes [23]. Chen and Zhi-Min [24] reported that balanced K^+^, Ca^2+^, and Mg^2+^ are important for plant survival at the salt-stressed condition. 

Conversely, Gurmani et al. [25], Rahman et al. [2], Hossain et al. [3], and Zhou et al. [23] showed the plant tolerance by external use of hormone, nutrient, organic acid, and antioxidants respectively through balancing ionic homeostasis. Therefore, similar results were obtained in the present study by exogenous Qu application. Quercetin-induced higher Ca^2+^ content might be the reason for lower Na^+^ accumulation. Similarly, COU reduced Na^+^ content in wheat along with increased K^+^ content under salt stress by modulated ion selectivity [19]. Mekawy et al. [20] also reported that apigenin application decreased Na^+^/K^+^ ratio in salt-stressed rice.

Salt-induced osmotic stress negatively affects water uptake and photosynthesis of plants [26], indicated by higher osmotic potential and lower LRWC [2], which is also evident from our study. Ahmed et al. [17] also found lower LRWC in salt-stressed tomato, which might be attributed to salt-induced root damage for which plants suffered from water shortage [27]. Conversely, Pro acts as cell osmoprotectant for osmotic adjustment, ROS scavenging, macromolecule stabilizations to lessen stress-induced damages [28], moreover balance osmotic potential and leaf expansion [29]. Ahmed et al. [17] reported stress-induced higher Pro accumulation in tomato with lowered RWC, which supports our results. However, Qu-induced increased Pro accumulation along with higher LRWC under salinity, is speculated to alleviate salt-induced osmotic stress, which is supported by Saleh and Madany [19] in COU-treated wheat seedlings under salt stress. They also observed that COU-induced higher Pro was owing to the increased pyrroline-5-carboxylate synthase (P5CS) activity and/or inhibited proline dehydrogenase (PDH) activity [30,31]. Thus, Qu alleviates salt-induced osmotic stress through upregulation of Pro synthesis.

Besides ionic and osmotic stresses, plants suffered from oxidative damage and subsequent cell death by upsetting cellular metabolism [18], which is in line with our study. Likely, Manai et al. [32] found higher ROS with elevated MDA content in salt-stressed tomato. Salt-stress-induced higher ROS and LOX activity which hamper cell membrane integrity in plants [2], which is evident from EL data in our study as an indication of membrane damage. Moreover, salt-treated higher root EL might be the reason for root damage and consequent osmotic suffering. However, Qu application alleviated ROS accumulation in tomato seedlings reduced oxidative damage by reducing LOX activity, MDA content, and EL. The involvement of phenolic compound to diminish stress-induced excess ROS production was reported by Mekawy et al. [20]. Hossain et al. [3] demonstrated the reduction of ROS alleviates the oxidative-stress-induced damage. Apigenin also significantly reduced the accumulation of ROS and lipid peroxidation in salt-treated rice [20].

Afterward, to understand the Qu-induced alleviation of oxidative stress, we explored the Qu-mediated antioxidant defense mechanism in salt-stressed tomato to know how Qu regulated both enzymatic and non-enzymatic antioxidants for scavenging excess ROS. To detoxify stress-induced toxic ROS, plants naturally exploit their antioxidant defense mechanism by exerting both non-enzymatic and enzymatic antioxidant components simultaneously. These antioxidants work in a systematic and cyclic way to diminish excess ROS, where SOD activity converts O_2_^•−^ to H_2_O_2_ as a first step defense [4]. We found higher SOD activity in tomato under salinity as an indication of higher O_2_^•−^ dismutation. Similarly, Ahmad et al. [17] reported higher SOD activity in salt-stressed tomato. Interestingly, in our study, Qu-treated tomato showed downward SOD activity under salinity, which might be due to less O_2_^•−^ production. Moreover, Qu is a potent antioxidant, which might be participating in ROS scavenging, as well as lowering the O_2_^•−^ production and thus reducing SOD activity.

Afterward, CAT activity was also increased and indicated salt-stress induced the excess H_2_O_2_. Likewise, increased CAT activity was also found in salt-affected lentil [3]. Moreover, upregulation of ROS scavenging enzymes (CAT, SOD, and POD) by using different phenolic compounds—like ellagate, ferulate, and cinnamate, respectively—have been reported under different stress conditions [33,34,35]. However, in our study, Qu supplementation to salt-stressed tomato seedlings decreased CAT activity, which might be attributed to the decrease of H_2_O_2_ content, as well as upregulation of AsA-GSH cycle.

In this AsA-GSH cycle, H_2_O_2_ is scavenged by the direct involvement of AsA and indirect participation from GSH. In the cell, AsA and GSH are used as a substrate for the enzymes APX and GPX and/or GST, respectively for ROS detoxification. During this process, DHA and GSSG were produced from AsA and GSH, respectively and at the end; AsA and GSH are regenerated by other enzymes.

From our study, we found lower contents of both AsA and GSH along with the increased activity of APX, GPX, and GST under salinity. Moreover, the contents of DHA and GSSG were higher, which is correlated with the involvement of both AsA and GSH to scavenge excess H_2_O_2_. As a result, both of AsA/DHA and GSH/GSSG ratios were reduced under salt stress. Ahmad et al. [17] also found increased APX and GPX activity along with lowered AsA in salt-stressed tomato, which corroborates our study. However, they observed increased GSH content in salt-affected tomato, which runs counter to our findings. However, under non-stressed conditions, Qu reduced the AsA/DHA ratio with lower AsA content which might be correlated with Qu-induced higher APX activity, interestingly, DHA content was not increased; moreover, DHAR activity decreased in Qu treated non-stressed seedlings. This might be because of the further degradation of DHA into other organic acids [4]. Conversely, Qu application increased AsA and GSH contents, and decreased APX and GPX activities along with lowered H_2_O_2_ in salt-stressed seedlings, which might be attributed to Qu induced reduction of H_2_O_2_, for which APX and GPX activities reduced, creating a lower requirement of AsA and GSH. In opposition, Apigenin increased APX activity in salt-stressed rice with the removal of toxic H_2_O_2_ [20]. In addition, exogenous Qu application improved both AsA/DHA and GSH/GSSG redox ratios under salt stress, which revealed the Qu-induced lower ROS production.

Under salinity, higher DHAR activity assists in recycling DHA to AsA and thus converts GSH to GSSG [2]. Again, stress-induced higher MDHAR activity recycles AsA from MDHA with assistance from NADPH [4]. Although MDHAR activity increased under salt stress, but decreased AsA content might be due to higher APX activity. Moreover, increased DHAR activity tried to mitigate the scarcity of AsA, thus increasing GSSG content. Qu induced increased MDHAR activity thus smoothens AsA recycling, which supports Qu-mediated decreased DHAR activity. Again, GSSG is recycled back to GSH by NAPDH dependent GR activity [4]. Although salt stress caused higher GR activity and we observed lower GSH content, which indicates the increased utilization of GSH. Manai et al. [32] and Ahmad et al. [17] also reported the increased GR activity in salt-treated tomato. However, Qu-treatment showed a further increment of GR activity under salt stress, resulting in lowered GSSG content supporting Qu-induced higher GR activity. Moreover, the decrease in GPX activity might be another cause for decreasing GSSG content in Qu-supplemented salt-stressed seedlings.

Furthermore, the GST activity not only takes part in scavenging H_2_O_2_ by using GSH but also has xenobiotic detoxification properties, by which they detoxify some endogenous toxic substance upon stress [4]. Therefore, Qu-induced higher GST activity is actively involved in increasing salt tolerance. Saleh and Madany [19] also found COU-induced higher total antioxidant capacity of wheat as well as increased salt tolerance by accumulating higher cellular antioxidants. Abu El-Soud et al. [33] also supported this.

Plants also possess glyoxalase system—including GSH dependent Gly I and Gly II—to detoxify cytotoxic MG, which is highly accumulated in cells due to abiotic stress [4]. Salinity increased MG content in tomato seedlings instead of higher activities of both Gly I and Gly II. Rahman et al. [2] and Nahar et al. [36] separately found the similar results in salt-stressed rice and mung bean, respectively. Many previous reports suggested the exogenous protectant-induced stimulation of glyoxalase system as well as MG detoxification [2,4,29,37]. Exogenous Qu-induced higher activities of Gly I and Gly II and thus decreased MG content with a steady-state higher GSH content in stressed seedlings, thus regulating the glyoxalase system for increasing tolerance, which is corroborated in other studies [4,29].

Hence, our result suggests that Qu has a promising role in regulating antioxidant defense, glyoxalase systems, and mineral homeostasis in the alleviation of salt toxicity and governing growth improvement.

## 4. Materials and Methods

### 4.1. Growth of Seedling and Stress Treatment

Tomato (*Solanum lycopersicum* L. cv. Pusa Ruby) seeds were germinated on filter paper in Petri plates (9 cm diameter) in a germination chamber. After germination, seedlings were transferred to a growth chamber by keeping 10 seedlings per Petri plates under a controlled environment (temperature, 25 ± 2 °C; light, 350 μmol photon m^−1^s^−2^; and relative humidity, 65–70%). Full strength Hoagland nutrient solution [38] was supplied to nourish the seedlings. Then 10-d old seedlings were treated with salt (NaCl, 150 mM) and Qu (15 and 25 µM) in solely and in combination as a co-treatment. The respective salt treatment was incorporated with nutrient solution and renewed every day with and/or without Qu during the whole period of study. Just before application, 50 mM 1 mL Qu stock solution was prepared using absolute ethanol. Afterwards, required amount of Qu stock solution as per treatment was mixed with the nutrient solution. Control treated seedlings received neither salt not Qu; only nutrient solution. Data were collected from third and fourth leaves of tomato seedlings after 5 days of treatment. For clarification and validation, the experiment was executed three times. Each time there were three replications for each treatment. For data collection there were 10 seedlings. Moreover, morphological data was measured from 10 seedlings from each treatment and expressed from its average value. 

### 4.2. Determination of Seedling Growth and Biomass Accumulation

Seedling growth parameters (shoot height, root length, stem girth) were taken from 10 randomly selected seedlings immediately after treatment duration and expressed after making an average of them. Seedling biomass accumulation was evaluated by observing both fresh and dry weight of shoot and roots of those selected seedlings and their mean value was calculated. 

### 4.3. Determination of Photosynthetic Pigment Contents

Chlorophyll *a*, Chl *b*, and Car were measured following Wellburn [39] to evaluate the photosynthetic pigment contents. 

### 4.4. Determination of Na, K, Ca, and Mg Contents

Whole plants were collected, excised to separate root and shoot, and oven-dried separately at 70 °C for 72 h. Amount of 0.1 g from dried tissue was digested with HNO_3_:HClO_4_ (5:1) acid mixture according to Rahman et al. [2]. From digested solution Na, K, Ca, and Mg contents were observed at atomic absorption spectrophotometer (AA-7000, Shimadzu, Kyoto, Japan).

### 4.5. Determination of Osmotic Status in Leaves

Leaf relative water content (LRWC) was measured from third and fourth leaves of tomato by taking the fresh, dry, and turgid weight [40].

According to Bates et al. [41], the free Pro accumulation was measured spectrophotometrically at 520 nm and calculated by using a standard curve.

### 4.6. Histochemical Detection of H_2_O_2_ and O_2_^•−^

Salinity-induced oxidative stress was detected by histochemical localization of O_2_^•−^ and H_2_O_2_ generation in leaves tissue by staining with nitroblue tetrazolium chloride (NBT) and 3′,3′-diaminobenzidine (DAB) solution, respectively [42]. 0.01% of acidic NBT and DAB solution were used separately to dip the fresh leaf tips in two glass tube followed by incubation at 25 °C. About 12 h of incubation leaves were distained using 70% ethanol, followed by repeated washing by DH_2_O. Afterward, the blue and brown spots were observed for O_2_^•−^ and H_2_O_2_ production, respectively.

### 4.7. Quantification of Oxidative Stress Marker and Lipid Peroxidation

Salt-induced higher accumulation of H_2_O_2_ was measured according to Yang et al. [43] expressed as µmol g^−1^ FW.

To measure the lipid peroxidation, MDA content (nmol g^−1^ FW) was quantified spectrophotometrically from the absorbance of 532 and 600 nm by following the methods of Heath and Packer [44].

### 4.8. Determination of Electrolyte Leakage

Electrolyte leakage from leaves and roots tissue was estimated by following Dionisio-Sese and Tobita [45]. Collected 0.2 g sample was dipped into 20 mL DH_2_O containing glass tube covered with a cap by cutting it into small pieces (less than 1 cm) and incubating it at 35 °C for 1 h. The first electrical conductivity (EC, E1) was measured by an electrical conductivity meter. After that, the tubes were autoclaved for 20 min at 121 °C and the second EC (E2) was observed after cooling. Finally, EL was calculated by using the following equation, EL (%) = E1/E2 × 100. 

### 4.9. Estimation of Ascorbate and Glutathione Contents

Leaf samples were extracted according to Kampfenkel et al. [46] for quantifying AsA and GSH content. The extracted supernatant was neutralized 0.5 M with K-P buffer (pH 7.0) to measure AsA and GSH. Dithiothretitol (DTT; 0.1 M) was added to convert the DHA to AsA and then total and reduced AsA were assayed spectrophotometrically at 265 nm. For calculating AsA, a standard curve of AsA was used. While, DHA was calculated after subtracting reduced AsA from total AsA [47]. 

Based on enzymatic recycling, glutathione content was determined spectrophotometrically at 412 nm by using standard curves of known concentrations of GSH and GSSG [47]. Hence, 2-vinylpyridine was used to remove GSH for determining GSSG content and the GSH content was measured by subtracting the GSSG from total GSH. 

### 4.10. Protein Quantification

Protein was determined by UV–visible spectrophotometry at 595 nm where BSA was used as a protein standard [48].

### 4.11. Extraction and Assays of Enzymatic Activity

Extraction buffer containing K-P buffer (50 mM; pH 7.0), AsA (1 mM), KCl (100 mM), β-mercaptoethanol (5 mM) and glycerol (10%; w/v) was used by keeping on ice to homogenize and extract 0.5 g of leaves. The extraction sample was centrifuged (at 11,500× *g*; 4 °C) for 12 min to get clear supernatant and further used to estimate the activities of the enzymes.

Lipoxygenase (LOX; EC: 1.13.11.12) activity was determined by observing the increasing absorbance at 234 nm spectrophotometrically, where linoleic acid was used as a substrate [49].

Superoxide dismutase (SOD; EC: 1.15.1.1) activity was observed by following El-Shabrawi et al. [5]. The absorbance was taken at 560 nm from the reaction mixture of K–P buffer (pH 7.0), NBT (2.24 mM), catalase (0.1 units), xanthine oxidase (0.1 units), xanthine (2.36 mM, pH 7.0), and enzyme. The SOD activity expressed as U min^−1^ mg^−1^ protein where U is the amount of enzyme required to inhibit NBT reduction by 50%.

Catalase (CAT; EC: 1.11.1.6) activity was assayed by following Hasanuzzaman et al. [47]. The decreasing absorbance was recorded at 240 nm from the reaction between enzyme extract and reaction buffer prepared from 50 mM K-P buffer (pH 7.0) and 15mM H_2_O_2_.

Ascorbate peroxidase (APX, EC: 1.11.1.11) activity was determined at 290 nm from the reaction mixture of the enzyme, 50 mM K-P buffer (pH 7.0), 0.5 mM AsA, 0.1 mM H_2_O_2_, 0.1 mM EDTA, and enzyme [50].

Dehydroascorbate reductase (DHAR; EC: 1.8.5.1) activity was determined from the mixture of 50 mM K-P buffer (pH 7.0), 2.5 mM GSH, 0.1 mM DHA, 0.1 mM EDTA, and enzyme extract [29]. The increase of absorbance was recorded at 265 nm.

Monodehydroascorbate reductase (MDHAR; EC: 1.6.5.4) activity was assayed at 340 nm by following Nahar et al. [29]; where reaction mixture consisted of Tris-HCl buffer (50 mM, pH 7.5), AsA (2.5 mM), AO (0.5 units), NADPH (0.2 mM), and enzyme.

Glutathione reductase (GR; EC: 1.6.4.2) activity was observed from the decreasing absorbance of 340 nm [47]. The required reaction mixture was prepared with 0.1 M K-P buffer (pH 7.0), 1 mM GSSG, 1 mM EDTA, 0.2 mM NADPH, and enzyme. 

Glutathione *S*-transferase (GST; EC: 2.5.1.18) activity was observed at 340 nm from the reaction of GSH (1.5 mM), 1-chloro-2,4-dinitrobenzene (CDNB; 1 mM), and enzyme [47]. 

Glutathione peroxidase (GPX; EC: 1.11.1.9) activity was assayed at 340 nm as per described of Nahar et al. [29] by using the reaction mixture of K-P buffer (100 mM; pH 7.0), GSH (2 mM), EDTA (1 mM), NaN_3_ (1 mM), NADPH (0.12 mM), GR (1 unit), H_2_O_2_ (0.6 mM), and enzyme extract.

Glyoxalase I (Gly I; EC: 4.4.1.5) activity was observed at 240 nm for 1 min according to Hasanuzzaman et al. [47], where assay mixture consists of 100 mM K-P buffer (pH 7.0), 1.7 mM GSH, 15 mM MgSO_4_, 3.5 mM MG, and enzyme extract. 

Glyoxalase II (Gly II; EC: 3.1.2.6) activity was observed at 412 nm [51]. While 500 μL of the reaction mixture was prepared from 100 mM Tris-HCl buffer (pH 7.2), 0.2 mM DTNB, and 1 mM *S*-D-lactoylglutathione (SLG).

### 4.12. Estimation of Methylglyoxal Content

The MG accumulation was measured at 288 nm and calculated from a standard curve of known concentration [52].

### 4.13. Statistical Analysis

The observed data were evaluated statistically by using XLSTAT 2018 software [53] from three replications. Analysis of variance (ANOVA) technique was used for data analysis and comparison of the mean difference was done by Fisher’s least significant difference (LSD) test with a 5% level of significance.

## 5. Conclusions

In the present study on salinity induced toxicity, we investigated the exogenous Qu-induced salt tolerance in tomato seedlings. Seedlings suffered from growth and Chl reduction; as well as ionic, osmotic, and oxidative stress from salt exposure. Salinity also altered the enzymatic and non-enzymatic antioxidants. However, interestingly, Qu showed its protective effects against salt toxicity by better seedlings growth with maintaining higher photosynthetic pigments. Exogenous Qu in salt-treated seedlings induced upregulation of K^+^, Ca^2+^, and Mg^2+^ mineral content with lowered Na^+^ and higher Pro accumulation, which resulted in inhibition of ionic toxicity and osmotic stress, respectively. Quercetin application also stimulated ROS scavenging attributes by upregulating both enzymatic and non-enzymatic antioxidants. Exogenous Qu also enhanced MG detoxification, by which seedlings got relief from MG-induced toxicity, which suggested Qu-induced better physiological and biochemical activities. Therefore, this study demands further comprehensive research to explore endogenous Qu synthesis along with mineral homeostasis and signaling approach for higher osmoregulation with antioxidant defense and glyoxalase systems. Besides, more research on the application of Qu to different crop species in various agro-ecological zones could be conducted for knowing its appropriate effectivity, in order to recommend it as a stress protector in farmers’ fields.
